# Predicting the progression of ophthalmic disease based on slit-lamp images using a deep temporal sequence network

**DOI:** 10.1371/journal.pone.0201142

**Published:** 2018-07-31

**Authors:** Jiewei Jiang, Xiyang Liu, Lin Liu, Shuai Wang, Erping Long, Haoqing Yang, Fuqiang Yuan, Deying Yu, Kai Zhang, Liming Wang, Zhenzhen Liu, Dongni Wang, Changzun Xi, Zhuoling Lin, Xiaohang Wu, Jiangtao Cui, Mingmin Zhu, Haotian Lin

**Affiliations:** 1 School of Computer Science and Technology, Xidian University, Xi’an, China; 2 State Key Laboratory of Ophthalmology, Zhongshan Ophthalmic Center, Sun Yat-sen University, Guangzhou, China; 3 School of Software, Xidian University, Xi’an, China; 4 Zhongshan School of Medicine, Sun Yat-sen University, Guangzhou, China; 5 School of Mathematics and Statistics, Xidian University, Xi’an, China; Soochow University Medical College, CHINA

## Abstract

Ocular images play an essential role in ophthalmology. Current research mainly focuses on computer-aided diagnosis using slit-lamp images, however few studies have been done to predict the progression of ophthalmic disease. Therefore exploring an effective approach of prediction can help to plan treatment strategies and to provide early warning for the patients. In this study, we present an end-to-end temporal sequence network (TempSeq-Net) to automatically predict the progression of ophthalmic disease, which includes employing convolutional neural network (CNN) to extract high-level features from consecutive slit-lamp images and applying long short term memory (LSTM) method to mine the temporal relationship of features. First, we comprehensively compare six potential combinations of CNNs and LSTM (or recurrent neural network) in terms of effectiveness and efficiency, to obtain the optimal TempSeq-Net model. Second, we analyze the impacts of sequence lengths on model’s performance which help to evaluate their stability and validity and to determine the appropriate range of sequence lengths. The quantitative results demonstrated that our proposed model offers exceptional performance with mean accuracy (92.22), sensitivity (88.55), specificity (94.31) and AUC (97.18). Moreover, the model achieves real-time prediction with only 27.6ms for single sequence, and simultaneously predicts sequence data with lengths of 3–5. Our study provides a promising strategy for the progression of ophthalmic disease, and has the potential to be applied in other medical fields.

## Introduction

Ocular images play a vital role in clinical diagnosis and individualized treatment schedule of ophthalmic diseases [1–3]. Much attention has been focused on creating a computer-aided diagnosis system based on the currently available images, and committed to the enhancement of the diagnostic accuracy and efficiency [4–6]. These studies can classify and grade the severity of the emerging ophthalmic diseases, however they are incapable of predicting the impending trend of ophthalmic diseases. Moreover, it is difficult to predict the progression of diseases for ophthalmologists during their clinical practice. Because this prediction process involves a comprehensive analysis and comparison of the re-examination results from multiple stages, which suffers from time-consuming, subjective and waste of excellent physician resources [7, 8]. However, this prediction and inference for ophthalmic disease is of great clinical significance for the prognosis management and risk control [8–10], which can help ophthalmologists to implement therapeutic schedule effectively and remind the patients of what needs to be prevented. Therefore, more research is urgently needed to explore a feasible and efficient strategy to predict the progression of ophthalmic diseases automatically and to provide appropriate treatment schedule in a timely manner.

**Fig 1:**
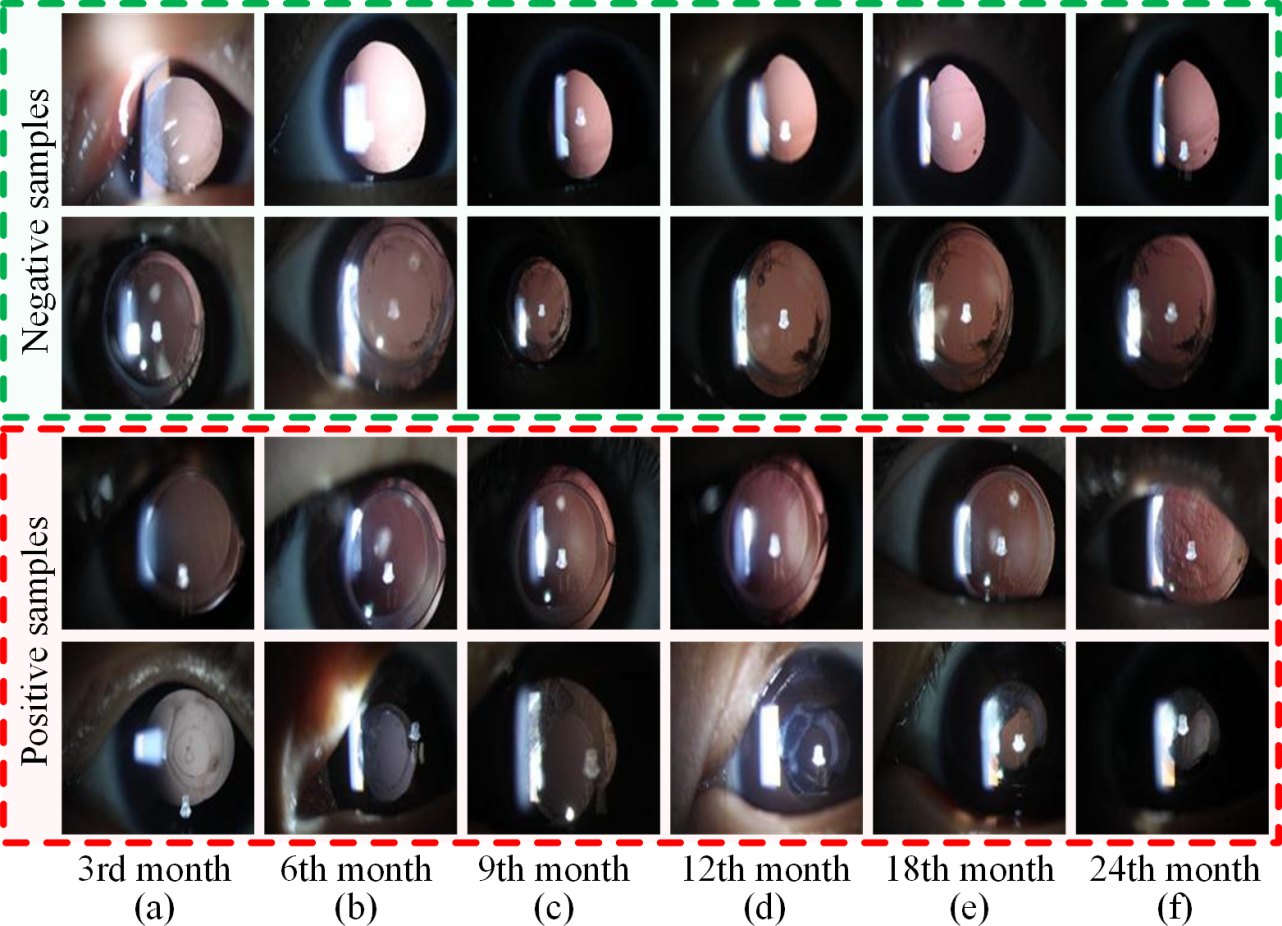
Examples of the progression of ophthalmic disease. **(a)–(f)** The slit-lamp images of six consecutive re-examination stages: the 3rd, 6th, 9th, 12th, 18th and 24th month. The first two rows are negative samples defined as manageable patients during the whole recovery period, while the third and fourth rows represent positive samples who require Nd-YAG laser surgery at the 6th re-examination stage. Notes: Nd-YAG: neodymium-doped yttrium aluminum garnet.

The number of blind people worldwide is projected to reach 75 million by the year 2020 [11]. Cataracts are the leading cause of blindness, accounting for about half of the blind globally [12]. Monitoring the recurrence of cataract surgery is a typical temporal sequence prediction scenario. Postoperative patient requires routine re-examination to monitor the changes of posterior capsular opacification and to gain favorable prognosis [13, 14]. During the re-examination process, we have accumulated a number of slit-lamp images, which include multiple sequential examination stages and form a complete temporal sequence dataset [15]. However, these images exhibit a variety of disease phenotypes, unavoidable noise, and its similarity or mutability between the before and after images [7, 16]. For example, as shown in Fig 1, the progression trend of the first three rows are stable from column a to f; whereas in the fourth row, the patient’s condition suddenly deteriorate from column b to c. The progression of cataract opacification is complicated in the slit-lamp sequence images, which cannot be simulated and predicted based on manually-designed features using simple linear models. These factors represent common problems of medical images and pose significant challenges for exploring an effective sequence method to predict the progression of ophthalmic diseases.

The current deep convolutional neural network (CNN) models have demonstrated extraordinary performance in image and video recognition tasks [17–19], especially in the automatic diagnosis of medical images [4, 6, 20, 21]. In previous works, we conducted extensive automatic diagnosis studies with satisfactory results for cataract and confirmed the effectiveness of high-level features extracted from the CNN model [6, 16, 22, 23]. In addition, long short term memory (LSTM) and recurrent neural network (RNN) models have achieved impressive performance in a wide variety of sequence learning and prediction tasks such as speech recognition [24], machine translation [25] and video understanding [26]. In this study, we propose an effective temporal sequence network (TempSeq-Net) to predict the progression of ophthalmic disease by combining deep CNN [19, 27, 28] and sequence processing method LSTM [29]. First, we employ a convolutional neural network to extract high-level features from the slit-lamp images, and then apply the LSTM method to mine their internal relations, so as to construct an end-to-end model to predict and analyze the progression of ophthalmic disease. Second, we conduct and compare six combinations of three CNNs and LSTM (or RNN) using 5-fold cross-validation to select the optimal combination. Third, we perform the detailed comparative experiments on different lengths of sequence data for training and prediction, evaluate their stability and validity, to determine the appropriate range of sequence lengths. Finally, we conclude the effective guidelines for the training and prediction of temporal sequence model in clinical application.

## Methods

### Ethics approval

The research protocol involving patients was approved by the Institutional Review Board/Ethics Committee of Xidian University and Zhongshan Ophthalmic Center of Sun Yat-sen University. The authors confirm that all methods were performed in accordance with the relevant guidelines and regulations. Written informed consent was obtained from all the study participants’ parents or legal guardian according to Childhood Cataract Program of the Chinese Ministry of Health (CCPMOH) [15].

### TempSeq-Net model

**Fig 2:**
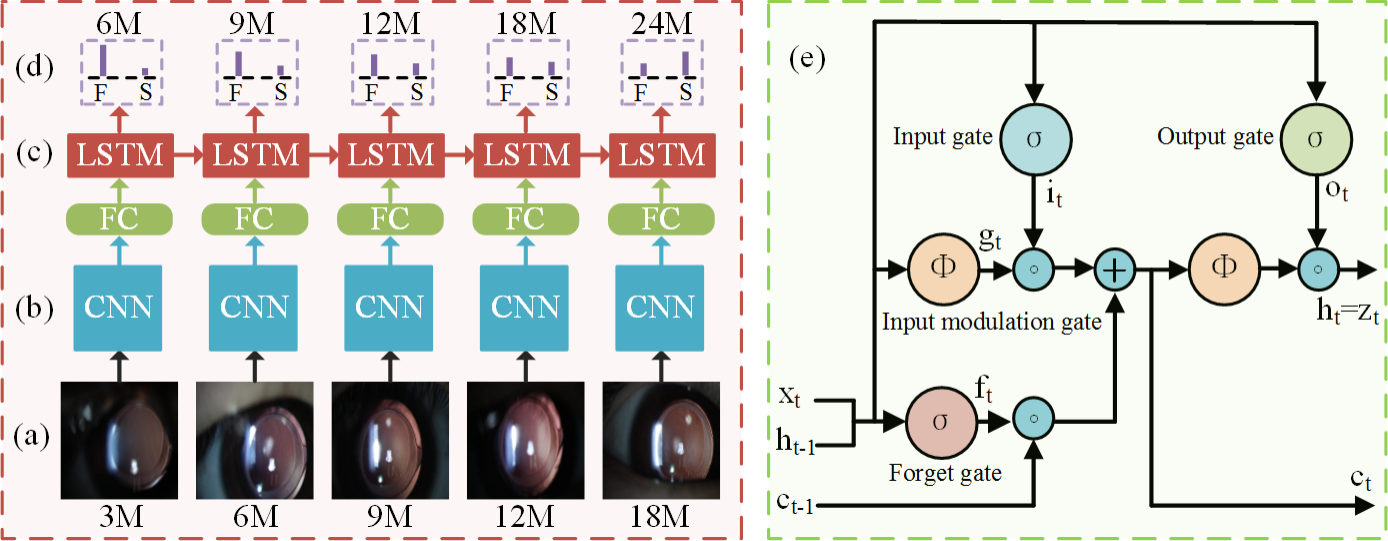
The architecture of the TempSeq-Net model. **(a)** Temporal sequence data inputs. The sequence images are sorted according to the re-examination stages and then entered into the convolutional neural network sequentially. **(b)** Convolutional neural network. The CNN is used for extracting the high-level features of temporal sequence images. **(c)** Long short term memory. The LSTM is used for mining and summarizing the internal rules of temporal sequence images. **(d)** The prediction output. The model predicts the probability of the progression of ophthalmic disease at an upcoming stage, where F and S represent follow-up and laser surgery, respectively. **(e)** The internal structure of the LSTM. Notes: 3M: the 3rd month of re-examination; FC: full-connected layer; TempSeq-Net: temporal sequence network.

As shown in Fig 2, the architecture of the TempSeq-Net mainly consists of temporal sequence data inputs (Fig 2A), convolutional neural network (CNN) (Fig 2B), long short term memory (LSTM) (Fig 2C) and prediction output (Fig 2D). The internal structure of the LSTM is shown in Fig 2E. In addition, the dataset augmentation and transfer learning are also essential technologies to overcome overfitting problem and accelerating model convergence. The technical details are described below.

To enhance the reproducibility of our proposed model and experiment results, we also deposited the TempSeq-Net model, source code, and training and evaluation procedures in dx.doi.org/10.17504/protocols.io.qgzdtx6.

### Convolutional neural networks

Three CNNs (AlexNet, GoogLeNet, and ResNet) are being compared to determine the best model for the sequence prediction tasks (Fig 2B). The AlexNet CNN [19] employed convolutional layers, overlapping pooling, fully-connected layers and non-saturating rectified linear units (ReLUs) to construct an eight-layer CNN, which won the first prize in the 2012 ImageNet Large Scale Visual Recognition Challenge (ILSVRC). Subsequently, a number variants of CNNs were presented to enhance model’s recognition performance. In particular, a 22-layer inception CNN (GoogLeNet) was proposed by Google researchers [27] based on the Hebbian principle, filter aggregation, average pooling and auxiliary classifier technologies. Kaiming He then employed residual connection scheme, batch normalization and scale operations to establish a 50-layer residual CNN (ResNet) [28]. Because these three CNNs perform distinct principles and demonstrate significant performance differences in the natural images recognition competition, we first need to measure their differences on the medical sequence prediction problems to select a better model.

### Long short term memory method

The RNN and LSTM have proven to be effective on sequence tasks [24–26] such as speech recognition, video understanding and text generation. Instead of using traditional RNN [30], the LSTM model [26] is adopted in this study because it provides a solution by incorporating memory unit to avoid the vanishing or exploding gradients problem during back-propagation. Benefited from the memory unit, the LSTM learns when to forget previous hidden states and when to update hidden states with the new information given. As shown in Fig 2E, the LSTM is updated at time *t* as Eq 1.

$$\begin{array}{l} i_{t}=\sigma (W_{xi}x_{t}+W_{hi}h_{t-1}+b_{i})\\ f_{t}=\sigma (W_{xf}x_{t}+W_{hf}h_{t-1}+b_{f})\\ o_{t}=\sigma (W_{xo}x_{t}+W_{ho}h_{t-1}+b_{o})\\ g_{t}=\phi (W_{xg}x_{t}+W_{hg}h_{t-1}+b_{g})\\ c_{t}=f_{t}\circ c_{t-1}+i_{t}\circ g_{t}\\ h_{t}=o_{t}\circ \phi (c_{t}) \end{array}$$ (1)

where *ϕ*(*x*) = (*e*^*x*^−*e*^−*x*^)/(*e*^*x*^+*e*^−*x*^) and *σ*(*x*) = (1+*e*^−*x*^)^−1^ are nonlinear functions, *x*_*t*_, *h*_*t*_, *i*_*t*_, *f*_*t*_, *o*_*t*_, *g*_*t*_, *c*_*t*_ and ∘ denote current input data, current hidden state, input gate, forget gate, output gate, input modulation gate, memory unit and element-wise product, respectively. The memory unit *c*_*t*_ is a function of the previous memory unit *c*_*t*−1_, the current input *x*_*t*_ and the previous hidden state *h*_*t*−1_. *f*_*t*_ and *i*_*t*_ enable the memory unit *c*_*t*_ to selectively forget its previous memory *c*_*t*−1_ or consider new input *x*_*t*_. These additional units enable the LSTM to learn very complex temporal dynamics for ophthalmic disease prediction.

### Transfer learning

Collecting a sample of ophthalmic sequence requires two-year follow-up from a patient after cataract surgery, so that the number of sequence samples is less than that of the natural images. It is insufficient to optimize millions of trainable parameters from scratch using the fully-trained method. In contrast, transfer learning [31, 32] is an alternative technology for medical images, which allows the pre-trained model to be fine-tuned from a better starting point and effectively accelerates the model’s convergence. First of all, we downloaded the trained model file of CNN from the caffe official website (https://github.com/BVLC/caffe/) and used it to initialize the parameters of the same layers of the TempSeq-Net. Then, we set the learning rate of the parameters of the Softmax classification layer and LSTM (or RNN) layer to 10 times that of other layers’ parameters. Therefore, this technology guaranteed that the parameters of these two new layers were fully trained while the parameters of other layers were only fine-tuned using the ophthalmic sequence images. The final trained model does not only inherit the color, texture and shape features of the natural images, but also learns the unique characteristics of ophthalmic sequence images. Furthermore, data augmentation methods including transformed images and horizontal reflections [33] are adopted to prevent over-fitting problem.

### Optimization process of the TempSeq-Net model

For one iterative training, *d* ophthalmic sequence samples are randomly selected to form a mini-batch training dataset $D=\{(x_{t}^{1},y_{t}^{1}{)}_{t=1}^{w},(x_{t}^{2},y_{t}^{2}{)}_{t=1}^{w},\ldots ,(x_{t}^{k},y_{t}^{k}{)}_{t=1}^{w},\ldots ,(x_{t}^{d},y_{t}^{d}{)}_{t=1}^{w}\}$. A sequence data $(x_{t}^{k},y_{t}^{k}{)}_{t=1}^{w}$ denotes consecutive *w* input data ($x_{t}^{k}$) and prediction label ($y_{t}^{k}$) of the *k*-th patient. The prediction label $y_{t}^{k}$ represents the progression trend of ophthalmic disease (laser surgery or follow-up) at time *t*+1. We optimize parameters of the TempSeq-Net model to minimize the cross-entropy loss function of a mini-batch sequence samples as shown in Eq 2.

$$J(\theta )=-\frac{1}{d}\left[\sum_{i=1}^{d}\sum_{t=1}^{w}\sum_{j=1}^{k}I\left\{y_{t}^{i}=j\right\}*\log\frac{e^{\theta_{j}^{T}x_{t}^{i}}}{\sum_{s=1}^{k}e^{\theta_{s}^{T}x_{t}^{i}}}\right]+\frac{\lambda }{2}\sum_{j=1}^{k}\sum_{s=1}^{m}\theta_{js}^{2}$$ (2)

where *d*, *t*, *k*, *m* and *θ* denote number of training sequence samples, time *t*, number of classes, number of input neurons, and trainable parameters respectively. $I\left\{y_{t}^{i}=j\right\}$ represents the indicator function ($I\left\{y_{t}^{i}\;is\;equal\;to\;j\right\}=1$ and $I\left\{y_{t}^{i}\;is\;not\;equal\;to\;j\right\}=0$). $\frac{\lambda }{2}\sum_{j=1}^{k}\sum_{s=1}^{m}\theta_{js}^{2}$ is a weight decay term which is applied to penalize larger trainable weights. We train the TempSeq-Net model using mini-batch gradient descent (Mini-batch-GD) [34], with back-propagation used to compute the gradient $\nabla_{\theta_{j}}J(\theta )$ over mini-batch *D* as Eq 3. Finally, we obtain the optimal trainable weights θ^*^ as Eq 4.

$$\nabla_{\theta_{j}}J(\theta )=-\frac{1}{d}\sum_{i=1}^{d}\left[\sum_{t=1}^{w}x_{t}^{i}*(I\{y_{t}^{i}=j\}-p(y_{t}^{i}=j|x_{1:t}^{i},y_{1:t-1}^{i},\theta ))\right]+\lambda \theta_{j}$$ (3)

$$\theta^{*}=\arg\;\underset{\theta }{\min}J(\theta )$$ (4)

## Results and discussion

### Dataset

A total of 6,090 slit-lamp images were derived from the Zhongshan Ophthalmic Center of Sun Yat-sen University [15, 35], the leading eye hospital in China. As shown in Fig 1, these images contain six consecutive re-examination stages (the 3rd, 6th, 9th, 12th, 18th and 24th month) from 1,015 patients with two years of follow-up. The positive samples (367) represented patients suffering from serious posterior capsular opacification (PCO) that required Nd-YAG (neodymium-doped yttrium aluminum garnet) laser treatment at the 6th re-examination stage, and the negative samples (648) are defined as manageable PCO patients during the whole recovery period. Each image was examined, discussed and labeled by three experienced ophthalmologists. More representative temporal sequence data of the slit-lamp images can be found in S1 File.

### Evaluation metrics

To evaluate the performance and stability of temporal sequence network (TempSeq-Net) for ophthalmic disease, we calculated six quantitative metrics, including accuracy (ACC), sensitivity (SEN), specificity (SPE), precision (PRE), F1-measure (F1_M), and G-mean (G_M), as follows.

$$\begin{array}{l} Accuracy=\left(TP+TN\right)/\left(TP+FN+TN+FP\right)\\ Sensitivity(Recall)=TP/\left(TP+FN\right)\\ Specificity=TN/\left(TN+FP\right)\\ Precision=TP/\left(TP+FP\right)\\ F1‑measure=(2\;*\;Recall\;*\;Precision)/(Recall+Precision)\\ G‑mean=\sqrt{\left(TP/(TP+FN))*(TN/(TN+FP)\right)} \end{array}$$ (5)

TP, FP, TN and FN represent the numbers of true positives, false positives, true negatives and false negatives respectively. The accuracy, sensitivity, specificity and precision are the common evaluation indicators for classification. Furthermore, the F1-measure, G-mean [36], the receiver operating characteristic curve (ROC), and the area under the ROC curve (AUC) indicators are employed to comprehensively measure the accuracies of the positive and negative samples at the same time.

### Overall prediction framework for the progression of ophthalmic disease

**Fig 3:**
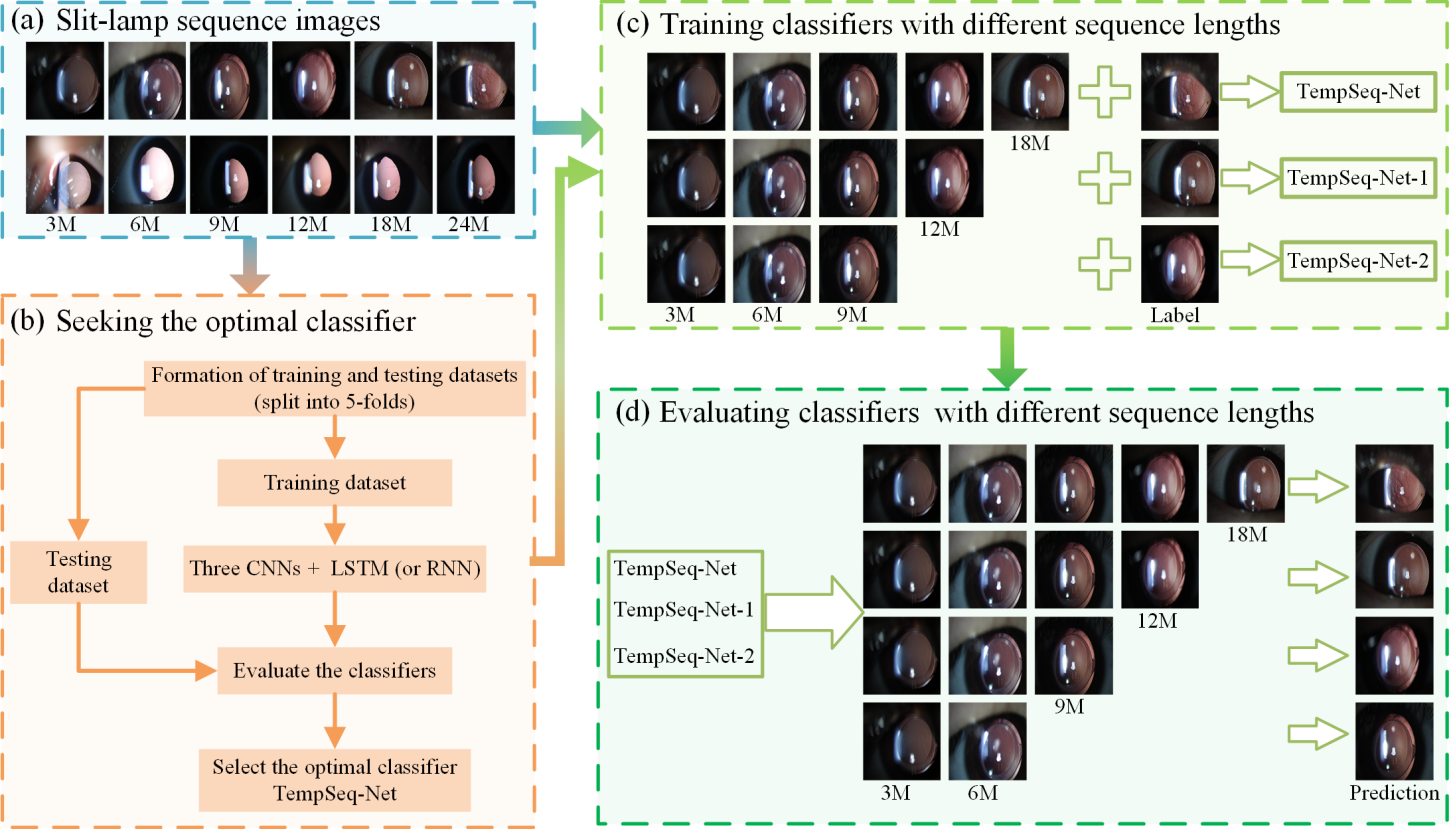
The overall prediction framework for the progression of ophthalmic disease. **(a)** The 6,090 slit-lamp sequence images consist of six consecutive re-examination stages (the 3rd, 6th, 9th, 12th, 18th and 24th month) of the 1,015 patients. Each image was examined and labeled independently by three experienced ophthalmologists. **(b)** Seeking the optimal classifier. The 5-fold cross-validation was employed to evaluate the performance of six combinations of three CNNs and two sequence methods (LSTM and RNN) to obtain the optimal TempSeq-Net model. **(c)** Training classifiers with different sequence lengths. Sequence datasets with different lengths (five, four and three) and their labels are employed to train three classifiers TempSeq-Net, TempSeq-Net-1 and TempSeq-Net-2, respectively. **(d)** Evaluating classifiers with different sequence lengths. The classifiers trained in the (c) are compared using sequence images with lengths of 2–5. Notes: CNN: convolutional neural network; LSTM: long short term memory; RNN: recurrent neural network.

As shown in Fig 3, the overall prediction framework consists of four modules: preparing the slit-lamp sequence images, seeking the optimal model TempSeq-Net, training and evaluating classifiers with different lengths of sequence images.

We employed a classification model to predict the progression of ophthalmic disease, of which the input images are the previous re-examination results and the label is the impending trend such as laser treatment or follow-up. First, the slit-lamp sequence images were resized to a size of 160×120 pixels and then sorted according to the re-examination time (Fig 3A). Second, we combined three CNNs (AlexNet, GoogleNet and ResNet) [19, 27, 28] and two sequence processing methods (LSTM and RNN) [29, 30] to construct six potential models. We trained these models with the first five images as input data and the 6th image as prediction label. We randomly divided the entire dataset into five equal parts and employed 5-fold cross-validation to fully evaluate the performance of these models in terms of effectiveness, efficiency and resource utilization to select the optimal model TempSeq-Net (temporal sequence network) (Fig 3B).

After obtaining the optimal model, we further explored the impact of different lengths of sequence data on the training and prediction procedure. Similarly, we employed two sequence datasets with different lengths (four and three) and their labels to train other two classifiers TempSeq-Net-1 and TempSeq-Net-2 respectively (Fig 3C), The results are compared with the TempSeq-Net classifier. Because the input data can be of arbitrary length in the prediction process, we evaluated these three classifiers using sequence data with lengths of 2–5 (Fig 3D) to help determine the appropriate range of sequence lengths for training and prediction.

### Experimental environment setting

In this study, all models were trained using four Nvidia Titan X graphics processing units based on the Caffe toolbox [37]. The mini-batch size was set to 25 on each GPU, to obtain 100 sequence data for one iteration training and calculated the average value of these samples to update the trainable parameters. The learning rate was initialized with 0.01 and successively reduced to one tenth of the original value per 500 iterations; a total of 2000 iterations were performed. Appropriate settings for these parameters can ensure rapid convergence and obtain better performance on ophthalmic sequence dataset. To facilitate research and reference, we also have released all source code of the TempSeq-Net model, which is available from Github: https://github.com/Ophthalmology-CAD/TempSeq-Net.

### Performance comparisons and optimal sequence model search

After applying 5-fold cross-validation, we calculated the detailed quantitative indicators with mean value and standard deviation, which included accuracy, specificity, sensitivity, AUC, F1-measure and G-mean to evaluate the performance of these six models (Table 1). From the experimental results, we obtained two meaningful conclusions. First, the LSTM method is better compared to the RNN method, which does not get effected when it is combined with any one of the CNNs. For example, the ACC, SEN, F1_M and G_M of GoogLeNet-RNN (85.71, 74.80, 78.83 and 82.78) are inferior to the GoogLnet-LSTM (92.51, 88.83, 89.50 and 91.67), and the similar results between LSTM and RNN are also showed on the Residual CNN. Although the differences between AlexNet-RNN and AlexNet-LSTM is not obvious, the SEN indicator of the LSTM method is enhanced by more than 2% compared to the RNN method. These performance improvements are mainly attributed to the fact that the LSTM method uses memory units to avoid the vanishing or exploding gradients problem existed in the RNN method. As the number of patient’s re-examination increases, the length of the image sequence becomes longer and the differences between LSTM and RNN would become more obvious. Second, the performance of three CNNs combined with LSTM is almost equivalent. The AlexNet and GoogLeNet is slightly better than the residual CNN (ResNet), this is mainly due to the limited medical images which is lacking to train ultra-deep ResNet.

**Table 1:** The quantitative evaluation of six different temporal sequence networks.

Method	ACC (%)	SPE (%)	SEN (%)	F1_M (%)	G_M (%)	AUC (%)
AlexNet-RNN	91.72(1.37)^§^	94.76(1.42)	86.26(2.73)	88.20(2.42)	90.40(1.70)	96.84(1.59)
GoogLeNet-RNN	85.71(3.75)	91.85(2.77)	74.80(8.70)	78.83(6.33)	82.78(5.14)	93.17(3.29)
ResNet-RNN	87.88(3.76)	93.48(1.78)	77.93(7.74)	82.19(5.49)	85.28(4.69)	94.74(1.91)
AlexNet-LSTM	92.22(1.98)	94.31(1.71)	88.55(3.32)	89.10(2.98)	91.38(2.24)	97.18(1.46)
GoogLeNet-LSTM	92.51(1.49)	94.62(0.93)	88.83(2.97)	89.50(2.36)	91.67(1.85)	97.04(1.08)
ResNet-LSTM	90.64(1.84)	94.60(0.46)	83.53(4.68)	86.45(3.19)	88.87(2.63)	96.11(1.84)

Notes: RNN: recurrent neural network; LSTM: long short term memory; ResNet: 50-layers residual neural network; AlexNet: eight-layers AlexNet neural network; GoogLeNet: 22-layers inception neural network; AlexNet-LSTM: the combination model of AlexNet neural network and LSTM; ACC: accuracy; SPE: specifcity; SEN: sensitivity; F1_M: F1-measure; G_M: G-mean; AUC: area under the receiver operating characteristic curve

§Mean (standard deviation).

**Fig 4:**
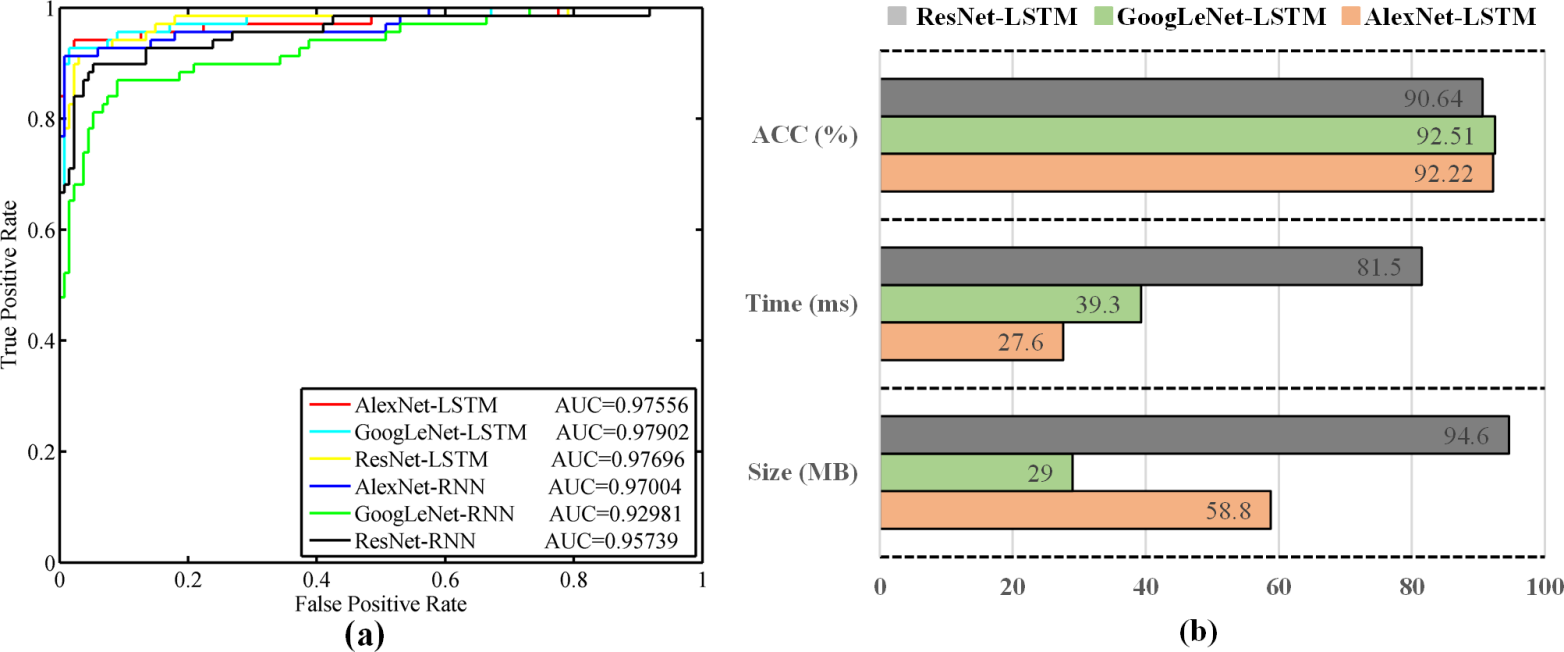
The ROC curves and performance comparison of six temporal sequence networks. **(a)** The ROC curves and AUC values of six temporal sequence networks: AlexNet-LSTM, GoogLeNet-LSTM, ResNet-LSTM, AlexNet-RNN, GoogLeNet-RNN and ResNet-RNN. **(b)** The performance comparison of LSTM models (AlexNet-LSTM, GoogLeNet-LSTM and ResNet-LSTM) in terms of accuracy, the model size and time consumption per sequence data. Notes: ROC: receiver operating characteristic curve.

Furthermore, we plotted the ROC curves to investigate the performance differences of the models (Fig 4A). The ROC curves of LSTM models are closer to the left upper corner than those of the RNN models, and all AUC indicators of LSTM models were maintained at above 0.975. This result also indicates that the LSTM models considerably outperform the RNN models in the prediction task of ophthalmic sequence data.

In addition, we also explored the linear classification model to predict the progression of ophthalmic disease. First of all, the color and texture features were extracted from the consecutive slit-lamp images based on our previous research [16, 22, 23], then we input them into the logistic regression classifier for prediction. However, the ACC, SPE, SEN, F1_M and G_M indicators of this model only reaches 73.79%, 82.10%, 58.94%, 62.10% and 69.54%, which is far weaker than the performance of the deep learning models. This is probably due to the fact that the relationship between temporal sequence data is not linear, especially in the real-world chaotic prediction of diseases' progression, and the linear classification model is not suitable for the prediction of ophthalmic disease. This result also further confirms the superiority and reasonability of the TempSeq-Net.

To select the best model from three LSTM models, we further compared their efficiency and resource utilization, including the model size, the number of parameters, the time consumption per sequence data, and GPU memory usage for testing. In this paper, we used the same sequence images, mini-batch size and hyper-parameters for fair comparison. We obtained the detailed comparison results as shown in the Table 2 and Fig 4B. The size and the number of parameters are the least for GoogLeNet-LSTM model, followed by the AlextNet-LSTM model. However, the AlexNet-LSTM (27.6ms) is faster than GoogleNet-LSTM (39.3ms) for single sequence prediction (Fig 4B), and the AlextNet-LSTM uses less GPU resource in testing procedure. ResNet-LSTM is inferior to the other two models in terms of accuracy, efficiency and resource usage. In general, the disk space of the computer is sufficient, but real-time prediction is required in clinical application. Therefore, we prefer AlexNet-LSTM as the final model (TempSeq-Net) and conduct further performance analysis based on this model.

**Table 2:** The efficiency and resource utilization comparison of three LSTM models.

Method	Size (MB)	Parameters	Time per sequence (ms)	GPU usage (MB)	Prediction accuracy
AlexNet-LSTM	58.8	1.5e+07	27.6	503	92.22
GoogLeNet-LSTM	29.0	7.6e+06	39.3	892	92.51
ResNet-LSTM	94.6	2.5e+07	81.5	3109	90.64

### Exploring effective range of sequence lengths for prediction

**Fig 5:**
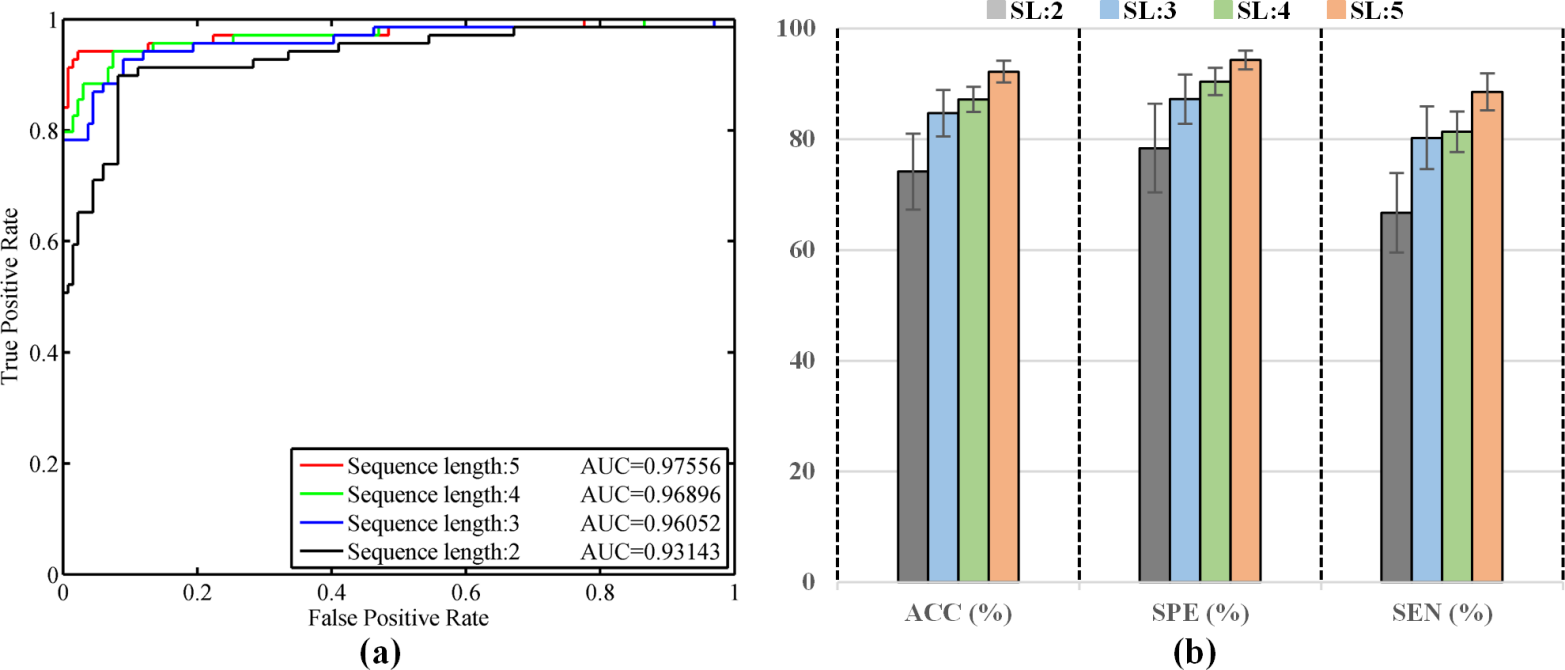
The performance comparison of TempSeq-Net model over different sequence lengths. **(a)** The ROC curves and AUC values of TempSeq-Net model over sequence lengths of 2–5. **(b)** The ACC, SPE and SEN indicators comparison of TempSeq-Net model over sequence lengths of 2–5. Notes: SL: sequence length.

Since the input data can be of arbitrary length, we want to explore the effect of different lengths of sequence images on the prediction performance to determine the appropriate range of sequence lengths for clinical use. We specifically selected and input four sequence data with different lengths (2, 3, 4, and 5 re-examination stages) into the TempSeq-Net model to predict their impending trend of ophthalmic disease at the next stage (3, 4, 5 and 6). Similarly, 5-fold cross-validation was employed to compare their differences in performance. We managed to achieve detailed quantitative indicators with means and standard deviations (Table 3), ROC curves (Fig 5A) and the histogram comparison of ACC, SPE and SEN (Fig 5B). From the experimental results, we obtained the following significant conclusions. First, when the length of sequence data is five, the prediction performance is the best with ACC, SPE, SEN and AUC achieving 92.22%, 94.31%, 88.55% and 97.18% (Table 3 and Fig 5B). Second, as the length of sequence data decreases, the prediction performance declines gradually (Fig 5B). Third, when the length is decreased to two, the performance is weak (only 74.19%, 78.42%, 66.73% and 91.47%) as shown in Table 3 and Fig 5B. Fourth, when the length is three or four, their results are almost comparable and slightly improved (84.73%, 87.25%, 80.27% and 94.59% for length three; 87.19%, 90.44%, 81.36% and 95.18% for length four). In addition, the ROC curves and AUC values declines when the sequence length reduces from five to two (Fig 5A). The experimental results indicate that our temporal sequence model TempSeq-Net can effectively predict the progression pattern of ophthalmic disease based on three or more consecutive re-examination results.

**Table 3:** The performance comparison of TempSeq-Net model for prediction with different sequence lengths.

SL	ACC (%)	SPE (%)	SEN (%)	F1_M (%)	G_M (%)	AUC (%)
2	74.19(6.86)^§^	78.42(8.01)	66.73(7.15)	65.27(8.22)	72.25(6.52)	91.47(1.52)
3	84.73(4.18)	87.25(4.42)	80.27(5.67)	79.08(5.95)	83.65(4.34)	94.59(1.14)
4	87.19(2.28)	90.44(2.48)	81.36(3.67)	82.04(3.68)	85.76(2.52)	95.18(1.30)
5	92.22(1.98)	94.31(1.71)	88.55(3.32)	89.10(2.98)	91.38(2.24)	97.18(1.46)

§Mean (standard deviation).

### Analyzing the impact of different sequence data lengths on model’s training

Furthermore, we want to analyze the impacts of different sequence data lengths on model’s training. We trained two other classifiers TempSeq-Net-1 and TempSeq-Net-2 using sequence lengths of four and three, respectively, and compare them with TempSeq-Net classifier trained with sequence length five (Table 4). For fair comparison, we conducted the same testing dataset in each group of comparative experiments. From the experimental results, we achieved a meaningful conclusion: the performance of the models trained with longer data performs better than or equal to that of the models trained with shorter data. For example, when the length of sequence data is three, although the ACC and SPE of three models are almost equal, the SEN, F1_M, G_M and AUC of TempSeq-Net (80.27, 79.08, 83.65 and 94.59), TempSeq-Net-1 (75.38, 76.01, 81.05 and 94.03) and TempSeq-Net-2 (74.01, 75.42, 80.49 and 93.73) are successively reduced. When the length of sequence data is four, the ACC, SPE and SEN indicators of the TempSeq-Net model (87.19, 90.44 and 81.36) are slightly better than those of the TempSeq-Net-1 model (87.09, 91.00 and 79.97) and TempSeq-Net-2 (82.86, 87.29 and 74.81). Similar results are also showed in the prediction of sequence data with length five, where the ACC, SPE and SEN indicators of TempSeq-Net (92.22, 94.31 and 88.55) is significantly better than that of the TempSeq-Net-1 classifier (87.68, 91.35 and 81.04). These experimental results indicate that training the model with longer sequence data can enhance its prediction performance. Only one model with longer sequence data needs to be trained to be able to simultaneously predict short and long sequence data. As the number of re-examination increases, the sequence data will become longer. Longer sequence data allows the model to obtain richer temporal-spatial relationship and present a more precise prediction for the progression of ophthalmic disease.

**Table 4:** The performance comparison of the models trained with different sequence lengths.

SL	Model	ACC (%)	SPE (%)	SEN (%)	F1_M (%)	G_M (%)	AUC (%)
3	TempSeq-Net	84.73(4.18)^§^	87.25(4.42)	80.27(5.67)	79.08(5.95)	83.65(4.34)	94.59(1.14)
	TempSeq-Net-1	82.96(3.86)	87.23(2.63)	75.38(6.74)	76.01(6.20)	81.05(4.72)	94.03(1.21)
	TempSeq-Net-2	82.76(4.78)	87.68(2.92)	74.01(8.55)	75.42(7.58)	80.49(5.90)	93.73(1.17)
4	TempSeq-Net	87.19(2.28)	90.44(2.48)	81.36(3.67)	82.04(3.68)	85.76(2.52)	95.18(1.30)
	TempSeq-Net-1	87.09(2.04)	91.00(1.96)	79.97(4.97)	81.66(3.30)	85.27(2.59)	95.24(1.39)
	TempSeq-Net-2	82.86(2.68)	87.29(2.80)	74.81(3.74)	75.90(3.85)	80.79(2.78)	93.77(1.18)
5	TempSeq-Net	92.22(1.98)	94.31(1.71)	88.55(3.32)	89.10(2.98)	91.38(2.24)	97.18(1.46)
	TempSeq-Net-1	87.68(2.06)	91.35(1.25)	81.04(4.23)	82.51(3.59)	86.02(2.64)	95.34(1.35)

Notes: TempSeq-Net-1: the temporal sequence network trained with sequence length four; TempSeq-Net-2: the temporal sequence network trained with sequence length three

§Mean (standard deviation).

### Convergence analysis of the TempSeq-Net model

**Fig 6:**
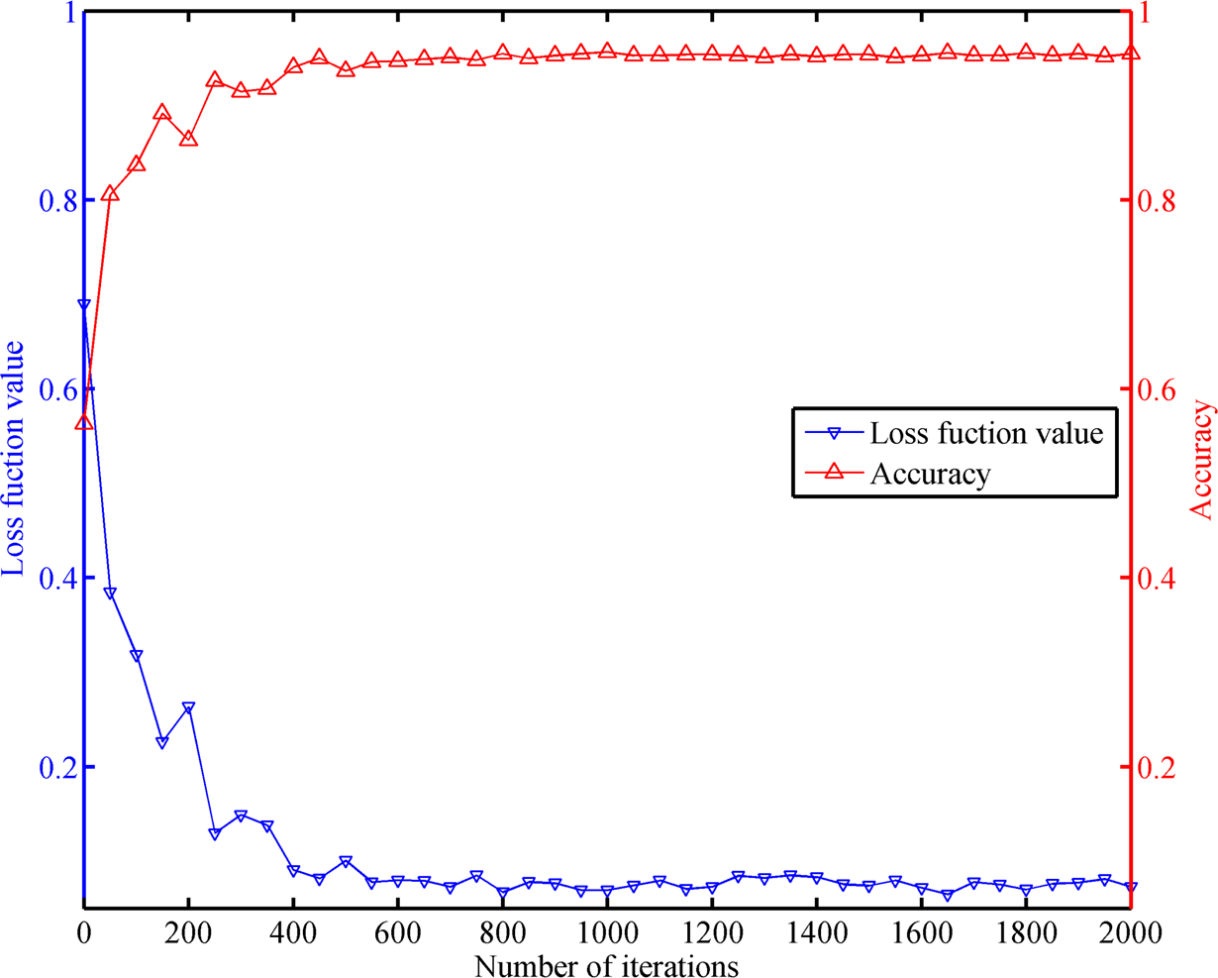
The convergence analysis of the TempSeq-Net model. The blue and red curves represent the changing trends of the loss function value and accuracy with iterations, respectively.

We also analyzed the convergence of the TempSeq-Net model under limited number of iterative training. We performed one testing per 50 training sessions and calculated its accuracy and its loss function value. A total of 2000 training sessions were conducted, we obtained 40 groups of accuracy and loss values. As shown in Fig 6, the loss function value and accuracy changed dramatically at the beginning of the training, however both of them tend to be stable with increasing iterations. This satisfactory performance indicates that our TempSeq-Net model is effective and convergent in the prediction of the ophthalmic diseases.

## Conclusions and future work

In this study, we proposed an effective and feasible temporal sequence network to predict the progression of ophthalmic disease based on the consecutive re-examination images. We have compared the performance of six different combinations of CNNs and LSTM (or RNN) under the same cross-validation dataset, to obtain the optimal TempSeq-Net model. Next, we evaluated the prediction effectiveness of the TempSeq-Net on different lengths of sequence data, and obtained the appropriate range of sequence lengths in prediction procedure. A meaningful conclusion was obtained that only one model needs to be trained for prediction with different sequence lengths. We also have achieved real-time prediction that can process single sequence data in tens of milliseconds. This approach provides a promising solution to this challenging task of ophthalmic disease prediction, which is of great benefit to the individual’s treatment schedule and as an early warning for ophthalmologists and patients. What’s more, our study opens up new possibility for artificial intelligence technologies in the prediction applications for other medical images, videos and electronical records.

In the future, we will develop and deploy a web-based software to serve ophthalmologists and patients, further validate the effectiveness of our approach in clinic, and gather more sequence data to enhance model’s performance. On the other hand, we will continue exploring different temporal sequence methods (such as Gated Recurrent Unit) to predict the progression of ophthalmic disease, and combine image localization (such as Faster RCNN or U-Net) and interpretable methods of deep learning to mine the relationship between disease progression and sequence images changes.

## Supporting information

S1 File Temporal sequence data of the slit-lamp images. (ZIP)
