# Supplementary figures and images for: Predicting the progression of ophthalmic disease based on slit-lamp images using a deep temporal sequence network
Source: PLoS One. 2018 Jul 31;13(7):e0201142. doi: 10.1371/journal.pone.0201142 (PMC6067742; doi:10.1371/journal.pone.0201142)

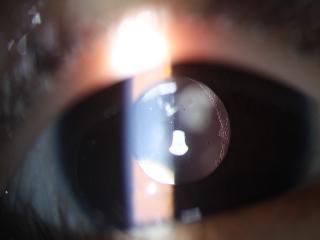

Supplement: S1 File — (ZIP) [file pone.0201142.s001.zip › S1_file/0001/0001_1_relapse.jpg]

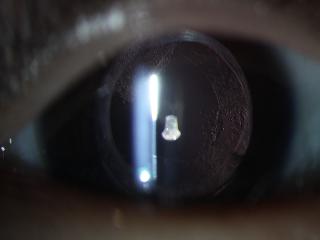

Supplement: S1 File — (ZIP) [file pone.0201142.s001.zip › S1_file/0001/0001_2_relapse.jpg]

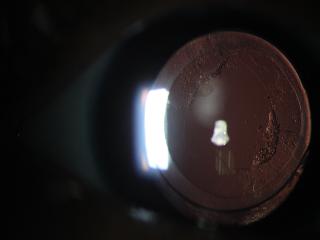

Supplement: S1 File — (ZIP) [file pone.0201142.s001.zip › S1_file/0001/0001_3_relapse.jpg]

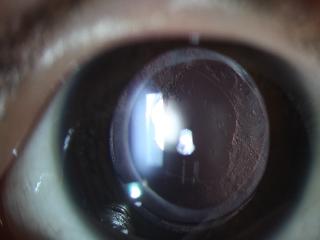

Supplement: S1 File — (ZIP) [file pone.0201142.s001.zip › S1_file/0001/0001_4_relapse.jpg]

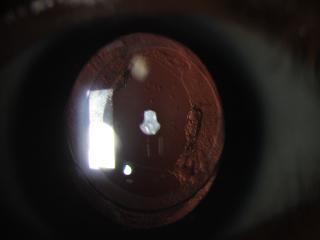

Supplement: S1 File — (ZIP) [file pone.0201142.s001.zip › S1_file/0001/0001_5_relapse.jpg]

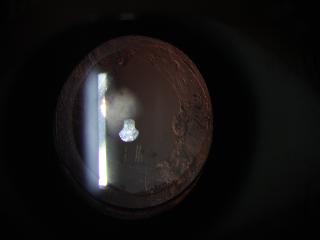

Supplement: S1 File — (ZIP) [file pone.0201142.s001.zip › S1_file/0001/0001_6_relapse.jpg]

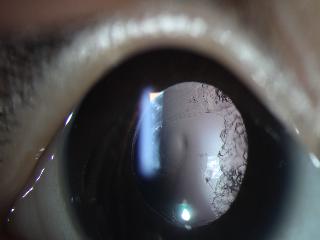

Supplement: S1 File — (ZIP) [file pone.0201142.s001.zip › S1_file/0002/0002_1_relapse.jpg]

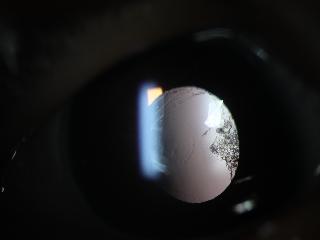

Supplement: S1 File — (ZIP) [file pone.0201142.s001.zip › S1_file/0002/0002_2_relapse.jpg]

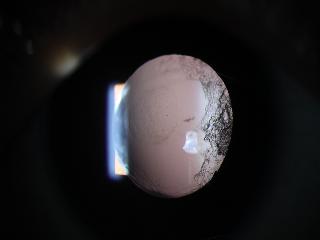

Supplement: S1 File — (ZIP) [file pone.0201142.s001.zip › S1_file/0002/0002_3_relapse.jpg]

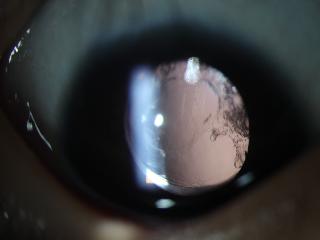

Supplement: S1 File — (ZIP) [file pone.0201142.s001.zip › S1_file/0002/0002_4_relapse.jpg]

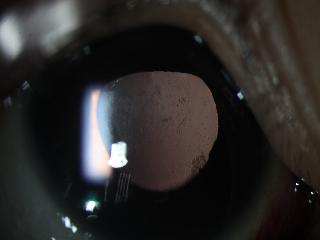

Supplement: S1 File — (ZIP) [file pone.0201142.s001.zip › S1_file/0002/0002_5_relapse.jpg]

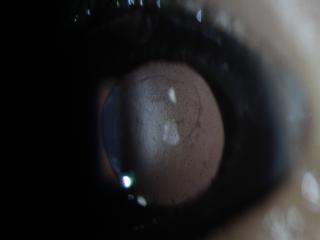

Supplement: S1 File — (ZIP) [file pone.0201142.s001.zip › S1_file/0002/0002_6_operation.jpg]

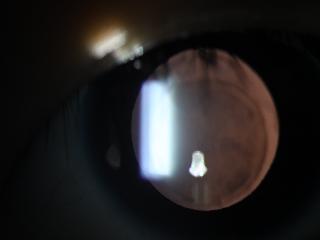

Supplement: S1 File — (ZIP) [file pone.0201142.s001.zip › S1_file/0003/0003_1_relapse.jpg]

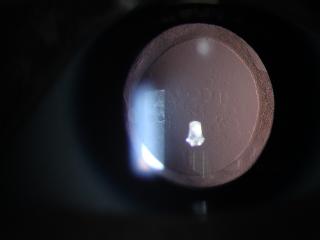

Supplement: S1 File — (ZIP) [file pone.0201142.s001.zip › S1_file/0003/0003_2_relapse.jpg]

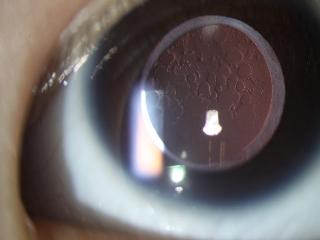

Supplement: S1 File — (ZIP) [file pone.0201142.s001.zip › S1_file/0003/0003_3_relapse.jpg]

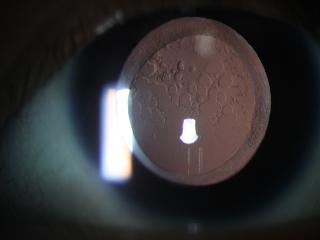

Supplement: S1 File — (ZIP) [file pone.0201142.s001.zip › S1_file/0003/0003_4_relapse.jpg]

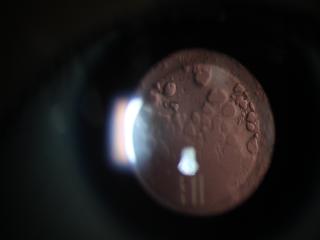

Supplement: S1 File — (ZIP) [file pone.0201142.s001.zip › S1_file/0003/0003_5_relapse.jpg]

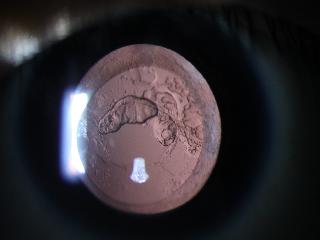

Supplement: S1 File — (ZIP) [file pone.0201142.s001.zip › S1_file/0003/0003_6_operation.jpg]

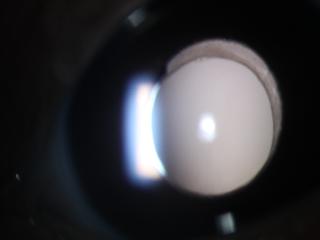

Supplement: S1 File — (ZIP) [file pone.0201142.s001.zip › S1_file/0004/0004_1_relapse.jpg]

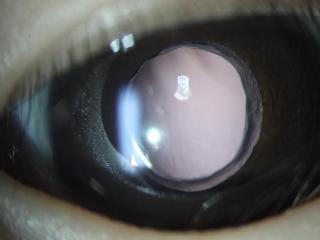

Supplement: S1 File — (ZIP) [file pone.0201142.s001.zip › S1_file/0004/0004_2_relapse.jpg]

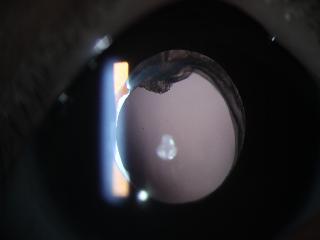

Supplement: S1 File — (ZIP) [file pone.0201142.s001.zip › S1_file/0004/0004_3_relapse.jpg]

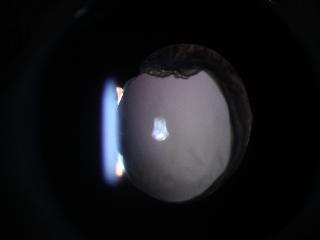

Supplement: S1 File — (ZIP) [file pone.0201142.s001.zip › S1_file/0004/0004_4_relapse.jpg]

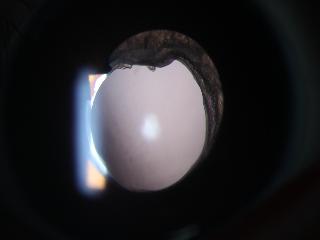

Supplement: S1 File — (ZIP) [file pone.0201142.s001.zip › S1_file/0004/0004_5_relapse.jpg]

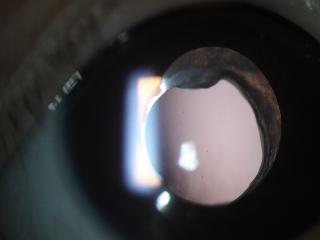

Supplement: S1 File — (ZIP) [file pone.0201142.s001.zip › S1_file/0004/0004_6_relapse.jpg]

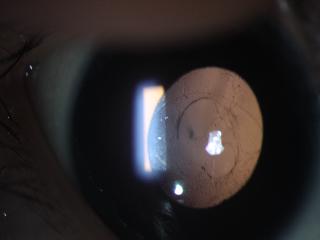

Supplement: S1 File — (ZIP) [file pone.0201142.s001.zip › S1_file/0005/0005_1_relapse.jpg]

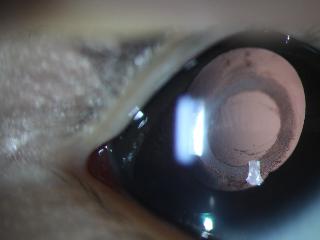

Supplement: S1 File — (ZIP) [file pone.0201142.s001.zip › S1_file/0005/0005_2_relapse.jpg]

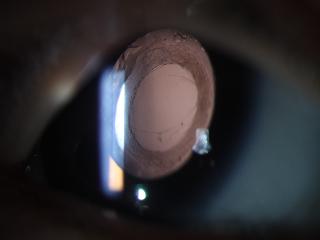

Supplement: S1 File — (ZIP) [file pone.0201142.s001.zip › S1_file/0005/0005_3_relapse.jpg]

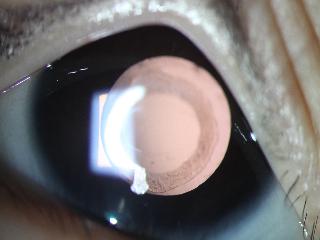

Supplement: S1 File — (ZIP) [file pone.0201142.s001.zip › S1_file/0005/0005_4_relapse.jpg]

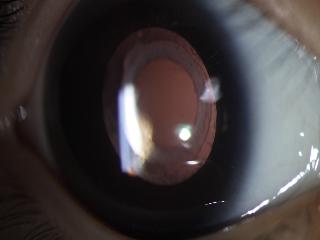

Supplement: S1 File — (ZIP) [file pone.0201142.s001.zip › S1_file/0005/0005_5_relapse.jpg]

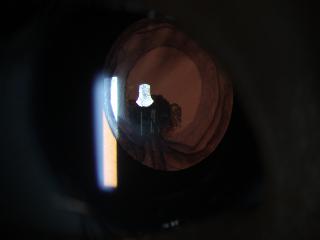

Supplement: S1 File — (ZIP) [file pone.0201142.s001.zip › S1_file/0005/0005_6_operation.jpg]

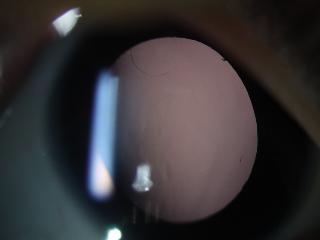

Supplement: S1 File — (ZIP) [file pone.0201142.s001.zip › S1_file/0006/0006_1_relapse.jpg]

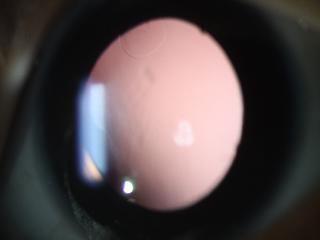

Supplement: S1 File — (ZIP) [file pone.0201142.s001.zip › S1_file/0006/0006_2_relapse.jpg]

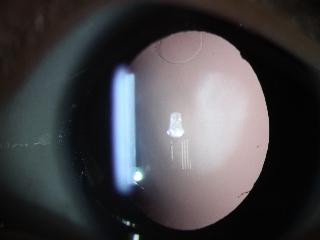

Supplement: S1 File — (ZIP) [file pone.0201142.s001.zip › S1_file/0006/0006_3_relapse.jpg]

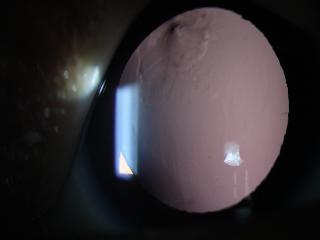

Supplement: S1 File — (ZIP) [file pone.0201142.s001.zip › S1_file/0006/0006_4_relapse.jpg]

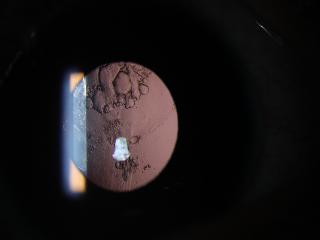

Supplement: S1 File — (ZIP) [file pone.0201142.s001.zip › S1_file/0006/0006_5_relapse.jpg]

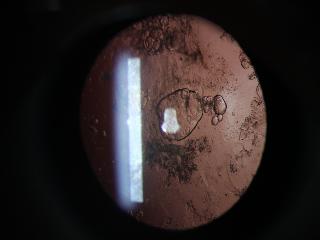

Supplement: S1 File — (ZIP) [file pone.0201142.s001.zip › S1_file/0006/0006_6_operation.jpg]

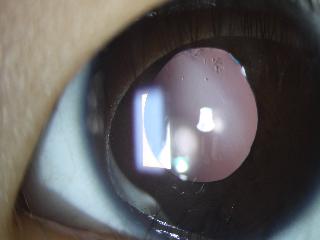

Supplement: S1 File — (ZIP) [file pone.0201142.s001.zip › S1_file/0007/0007_1_relapse.jpg]

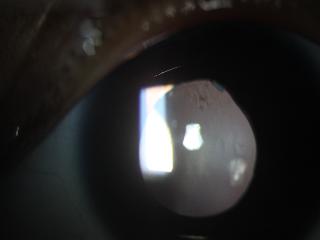

Supplement: S1 File — (ZIP) [file pone.0201142.s001.zip › S1_file/0007/0007_2_relapse.jpg]

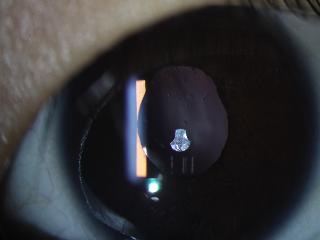

Supplement: S1 File — (ZIP) [file pone.0201142.s001.zip › S1_file/0007/0007_3_relapse.jpg]

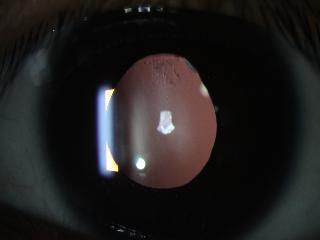

Supplement: S1 File — (ZIP) [file pone.0201142.s001.zip › S1_file/0007/0007_4_relapse.jpg]

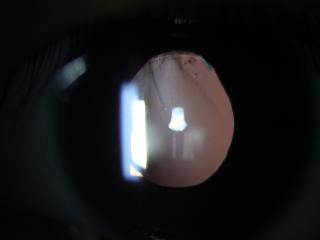

Supplement: S1 File — (ZIP) [file pone.0201142.s001.zip › S1_file/0007/0007_5_relapse.jpg]

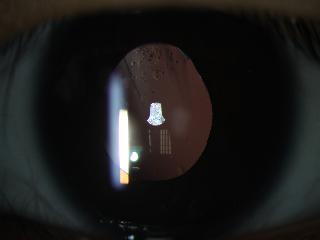

Supplement: S1 File — (ZIP) [file pone.0201142.s001.zip › S1_file/0007/0007_6_relapse.jpg]

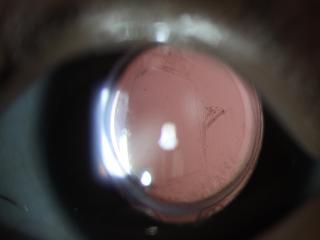

Supplement: S1 File — (ZIP) [file pone.0201142.s001.zip › S1_file/0008/0008_1_relapse.jpg]

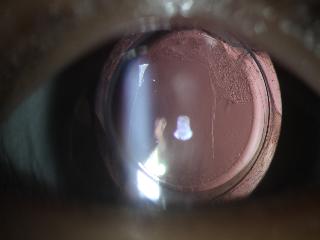

Supplement: S1 File — (ZIP) [file pone.0201142.s001.zip › S1_file/0008/0008_2_relapse.jpg]

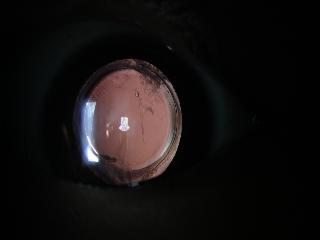

Supplement: S1 File — (ZIP) [file pone.0201142.s001.zip › S1_file/0008/0008_3_relapse.jpg]

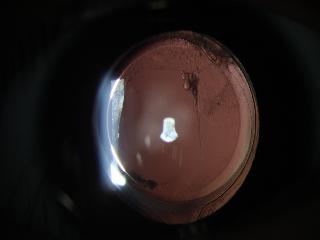

Supplement: S1 File — (ZIP) [file pone.0201142.s001.zip › S1_file/0008/0008_4_relapse.jpg]

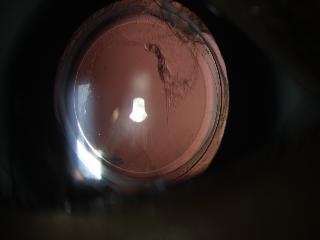

Supplement: S1 File — (ZIP) [file pone.0201142.s001.zip › S1_file/0008/0008_5_relapse.jpg]

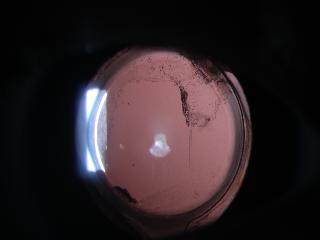

Supplement: S1 File — (ZIP) [file pone.0201142.s001.zip › S1_file/0008/0008_6_relapse.jpg]

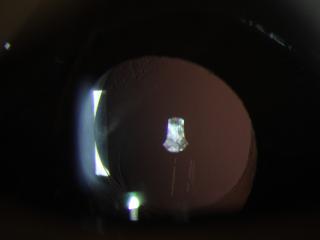

Supplement: S1 File — (ZIP) [file pone.0201142.s001.zip › S1_file/0009/0009_1_relapse.jpg]

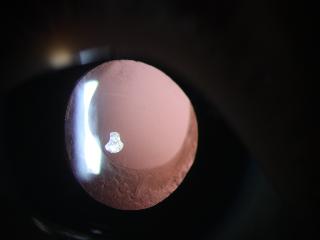

Supplement: S1 File — (ZIP) [file pone.0201142.s001.zip › S1_file/0009/0009_2_relapse.jpg]

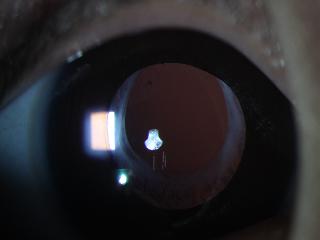

Supplement: S1 File — (ZIP) [file pone.0201142.s001.zip › S1_file/0009/0009_3_relapse.jpg]

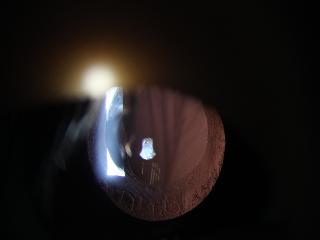

Supplement: S1 File — (ZIP) [file pone.0201142.s001.zip › S1_file/0009/0009_4_relapse.jpg]

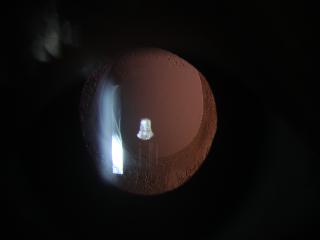

Supplement: S1 File — (ZIP) [file pone.0201142.s001.zip › S1_file/0009/0009_5_relapse.jpg]

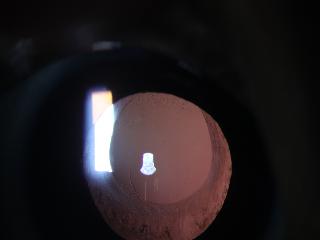

Supplement: S1 File — (ZIP) [file pone.0201142.s001.zip › S1_file/0009/0009_6_relapse.jpg]

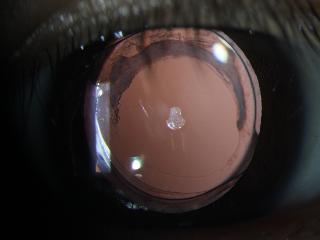

Supplement: S1 File — (ZIP) [file pone.0201142.s001.zip › S1_file/0010/0010_1_relapse.jpg]

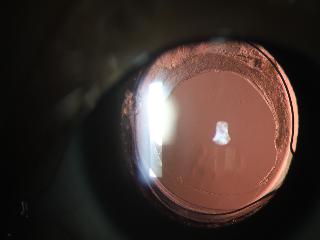

Supplement: S1 File — (ZIP) [file pone.0201142.s001.zip › S1_file/0010/0010_2_relapse.jpg]

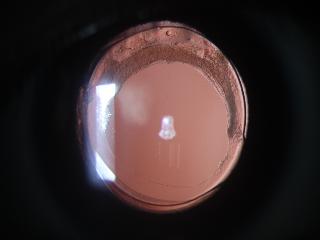

Supplement: S1 File — (ZIP) [file pone.0201142.s001.zip › S1_file/0010/0010_3_relapse.jpg]

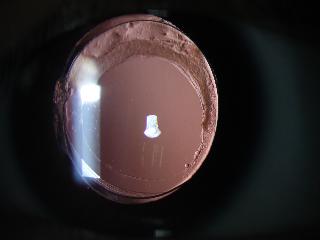

Supplement: S1 File — (ZIP) [file pone.0201142.s001.zip › S1_file/0010/0010_4_relapse.jpg]

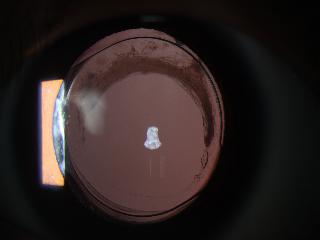

Supplement: S1 File — (ZIP) [file pone.0201142.s001.zip › S1_file/0010/0010_5_relapse.jpg]

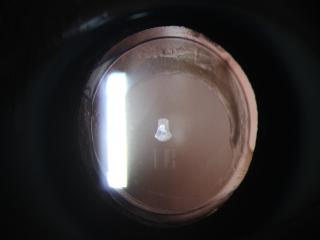

Supplement: S1 File — (ZIP) [file pone.0201142.s001.zip › S1_file/0010/0010_6_relapse.jpg]
